# The climate hazards infrared precipitation with stations—a new environmental record for monitoring extremes

**DOI:** 10.1038/sdata.2015.66

**Published:** 2015-12-08

**Authors:** Chris Funk, Pete Peterson, Martin Landsfeld, Diego Pedreros, James Verdin, Shraddhanand Shukla, Gregory Husak, James Rowland, Laura Harrison, Andrew Hoell, Joel Michaelsen

**Affiliations:** 1 US Geological Survey, Center for Earth Resources Observation and Science, 47914 252nd St., Sioux Falls, South Dakota 57198, USA; 2 UC Santa Barbara Climate Hazards Group, Santa Barbara, California 93106, USA; 3 National Oceanic and Atmospheric Administration Earth Systems Research Laboratory, Boulder, Colarodo 80305, USA

**Keywords:** Climate-change impacts, Hydrology, Environmental sciences, Attribution, Atmospheric dynamics

## Abstract

The Climate Hazards group Infrared Precipitation with Stations (CHIRPS) dataset builds on previous approaches to ‘smart’ interpolation techniques and high resolution, long period of record precipitation estimates based on infrared Cold Cloud Duration (CCD) observations. The algorithm i) is built around a 0.05° climatology that incorporates satellite information to represent sparsely gauged locations, ii) incorporates daily, pentadal, and monthly 1981-present 0.05° CCD-based precipitation estimates, iii) blends station data to produce a preliminary information product with a latency of about 2 days and a final product with an average latency of about 3 weeks, and iv) uses a novel blending procedure incorporating the spatial correlation structure of CCD-estimates to assign interpolation weights. We present the CHIRPS algorithm, global and regional validation results, and show how CHIRPS can be used to quantify the hydrologic impacts of decreasing precipitation and rising air temperatures in the Greater Horn of Africa. Using the Variable Infiltration Capacity model, we show that CHIRPS can support effective hydrologic forecasts and trend analyses in southeastern Ethiopia.

## Background & Summary

This paper describes the Climate Hazards group Infrared Precipitation with Stations (CHIRPS) environmental record (Data Citation 1), a new quasi-global (50°S-50°N), high resolution (0.05°), daily, pentadal, and monthly precipitation dataset. CHIRPS was developed to support the United States Agency for International Development Famine Early Warning Systems Network (FEWS NET). Building on approaches used in successful thermal infrared (TIR) precipitation products like the National Oceanic and Atmospheric Administration’s (NOAA’s) Rainfall Estimate (RFE2)^1,2^ and African Rainfall Climatology^3^ or the University of Reading’s TAMSAT African Rainfall Climatology And Time series (TARCAT)^4–6^, CHIRPS uses the Tropical Rainfall Measuring Mission Multi-satellite Precipitation Analysis version 7 (TMPA 3B42 v7)^7^ to calibrate global Cold Cloud Duration (CCD) rainfall estimates. Also building on approaches used in current state-of-the-science interpolated gauge products^8–12^, CHIRPS uses a ‘smart interpolation’ approach^13,14^, working with anomalies from a high resolution climatology. CHIRPS incorporates station data in a two phase process, producing two unique products. In the first phase, which yields a preliminary rainfall product with 2-day latency, sparse World Meteorological Organization’s Global Telecommunication System (GTS) gauge data are blended with CCD-derived rainfall estimates at every pentad. There are six pentads in a calendar month, five 5-day pentads and one pentad with the remaining 3 to 6 days of the month. Stations from Mexico are also included, because these data can be obtained in near real-time as well. In the second phase, which yields a final product with a ~3 week latency, the best available monthly (and pentadal) station data are combined with monthly (and pentadal) high resolution CCD-based rainfall estimates to produce fields that are similar to gridded monthly station products like those produced by the Global Precipitation Climatology Centre (GPCC)^8,12–14^ or University of East Anglia’s Climate Research Unit (CRU)^9,11^. Thus, the CHIRPS falls somewhere between heavily curated interpolated gauge datasets like the GPCC and sparse gauge plus satellite products like the RFE2.

At present, and on a global scale, there is an important gap in types of gridded precipitation datasets. There are datasets with a long period of record with very long latency, like the GPCC^8,12–14^ and CRU products^9,11^, and there are low latency precipitation estimates based solely on satellite information, like the TMPA 3B42 RT^7^, Climate Prediction Center MORPHing Technique (CMORPH)^15^, or Precipitation Estimation from Remotely Sensed Information using Artificial Neural Networks (PERSIANN)^16^ products, or on climate reanalysis systems, like the Coupled Forecast System (CFS) version 2 (ref. 17) or the European Centre for Medium-Range Weather Forecasts (ECMWF)^18^. The shortage of low latency, long record gridded data makes it challenging for scientists and analysts to place recent extremes in historic context. While one product (the Climate Prediction Center Merged Analysis of Precipitation (CMAP)^2^ blends station data and satellite estimates to produce a continuous 1979-present time series, it has a coarse 2.5° resolution. CHIRPS has been explicitly designed to fill this gap, providing blended gauge-satellite precipitation estimates that cover most global land regions and have a fairly low latency, high resolution, low bias, and long period of record. This dataset (as highlighted in our Usage Notes section) can be used in conjunction with land surface models to make effective mid-season drought forecasts or to analyze recent shifts in decadal precipitation in data sparse regions that depend on convective rainfall.

**Figure 1:**
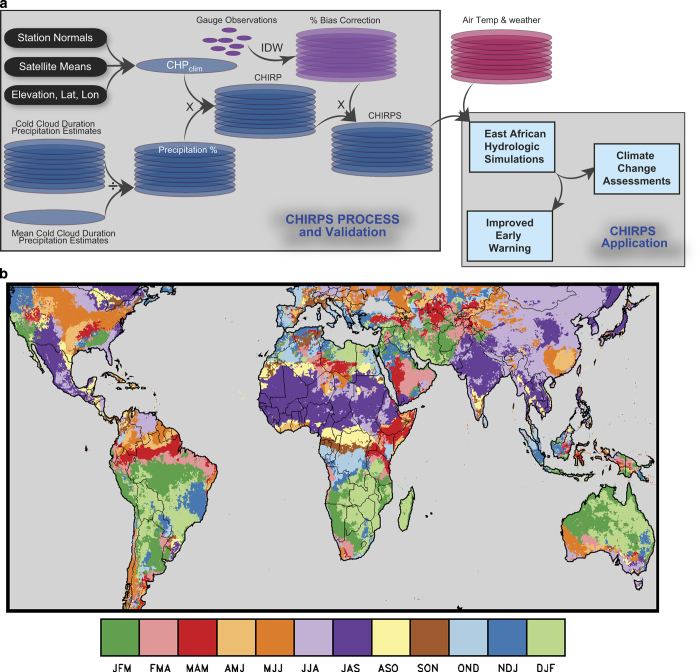
Overview of CHIRPS process and validation. (**a**) CHIRPS production and application schema. (**b**) Map showing the wettest three month seasons based on CHPclim.

Figure 1a shows the overall CHIRPS schema, as well as a summary of the example application we present in our Usage Notes section. The CHIRPS process involves three main components: i) the Climate Hazards group Precipitation climatology (CHPclim), ii) the satellite-only Climate Hazards group Infrared Precipitation (CHIRP), and iii) the station blending procedure that produces the CHIRPS. We describe the CHPclim, CHIRP, and CHIRPS and present validation analyses based on the GPCC and independent station datasets. We also provide a heuristic example showing how CHIRPS can be used in conjunction with hydrologic models to provide effective early warning of agricultural drought conditions and to assess the hydrologic impacts of low frequency changes in air temperatures and precipitation.

Figure 1b shows a map of our primary validation domain (126°W-90°E). While CHIRPS is quasi-global (50°S-50°N), and extends across all longitudes, we present validations over only part of this domain (126°W-153°E), to facilitate visualization, and because much of the remaining regions are ocean. Because CHIRPS has been primarily developed to support agricultural drought monitoring, we focus on evaluating performance during each location’s wettest precipitation season. In most regions with rain fed or irrigated crops, there is a strong dependency on precipitation totals during the peak months of precipitation.

Highlights:

- We present a new dataset, explicitly designed for monitoring agricultural drought and global environmental change over land.
- This new dataset allows us to place recent climate extremes in historical context.
- The dataset has spatial and temporal coverage, latency, and resolution that is unprecedented for a global terrestrial product.
- Validation results indicate good performance for drought monitoring.
- A sample application uses CHIRPS to monitor and predict post-1999 hydrologic anomalies over Ethiopia.
- This analysis identifies major drought contributions from both increasing air temperatures and decreasing precipitation.

## Methods

### CHPclim

For most regions of the world, there are more stations with historical long-term monthly means available than stations with consistent time varying monthly observations. The CHIRPS process leverages this historical data by building the CHIRP and CHIRPS based on a global 0.05° monthly precipitation climatology—the CHPclim. A recent publication details the CHPclim dataset^19^ and compares its accuracy to similar global products. Two sets of monthly historical long-term means were used to create the CHPclim. The first set was a collection of 27,453 monthly stations obtained from the Agromet Group of the Food and Agriculture Organization of the United Nations (FAO). The second set of 20,591 stations was taken from version two of the Global Historical Climate Network (GHCN)^20^. The more extensive FAO normals were used to build preliminary precipitation field estimates. The differences between this surface and GHCN 1980–2009 averages were then calculated, interpolated, and used to adjust the final monthly surfaces to a 1980–2009 time period.

The CHPclim is unique because in addition to the typical physiographic indicators used in most current climatologies (elevation, latitude and longitude), CHPclim also includes information from monthly long-term mean fields from five satellite products: Tropical Rainfall Measuring Mission 2B31 microwave precipitation estimates^7^, CMORPH microwave-plus-infrared based precipitation estimates^15^, monthly mean geostationary infrared brightness temperatures^21^, and land surface temperature estimates^22^. All products were resampled to a common 0.05° grid.

Instead of the thin plate spline approach used to produce the CRU^11^ and WorldClim^23^ climatologies, the CHPclim uses a moving window regression. For each grid cell a local regression is applied using latitude, longitude, and one-to-three additional predictors drawn from the satellite fields and elevation and slope. Different models were fit to 73 different tiles, with the number of predictors varying based on station density. Each tile represents a unique geographic domain (i.e., southern South America, the Caribbean, or Western Europe). The models were fit to the FAO climate normals, and the residuals from these FAO climate normals were interpolated using inverse distance weighting and added to the regression fields to make a preliminary estimate. The 1980–2009 GHCN station data were then used to estimate ratio biases at each station. These ratios were interpolated and used to produce a final CHPclim field representing 1980–2009 climate normals. Temporal resampling and smoothing of the 12 monthly mean fields were used to derive 72 pentadal mean fields.

### CHIRP

We next describe the derivation of the pentadal CHIRP fields. The CHIRP fields are variations from the CHPclim mean. The approach uses local calibration of satellite precipitation estimates with TIR CCD statistics, and was motivated by University of Reading efforts in Africa^24^ and more recently, the TARCAT project^5,6^. In the University of Reading local calibration approach, one uses historical daily rainfall data to i) define an optimal CCD temperature threshold for a given region, and ii) develop regression relationships translating the CCD values into estimates of precipitation in millimeters. Since temperature drops rapidly with atmospheric height, and geostationary satellites observe the top of clouds, CCD values are a measure of the amount of time a given pixel has been covered by high cold clouds. In order to achieve quasi-global coverage, we have adopted an approach that is similar, but simpler than the University of Reading procedure. The CHIRP estimation procedure uses a fixed CCD threshold of 235°K, as used in the Global Precipitation Index^25,26^, and calibrates the CCD regressions using 2000–2013 0.25° TMPA 3B42 pentadal precipitation. Note that this refers to the 3B42 with stations, not the 3B42 RT, which does not contain stations.

For each 0.25° grid cell, for each month, regression slopes and intercepts are derived using pentadal TMPA and TIR CCD data. The use of TMPA 3B42 training data (as opposed to gauge observations) may increase the intercept values, causing CHIRP to overestimate the number of rainy days. These monthly 0.25° slopes and intercepts are resampled to a 0.05° grid, and used to produce 1981-present pentadal precipitation estimates. Each pentadal precipitation estimate is then translated into a fraction of normal by dividing each grid cell’s value by that grid cell’s 1981–2013 mean precipitation estimate. This fraction, multiplied against the corresponding CHPclim value, produces the CHIRP estimates. In this way, the CCD data are used to estimate variations around the CHPclim mean, which reduces the CHIRP systematic bias. The primary computing time step for the CHIRP is the pentad. All other time steps are either aggregates (dekadal and monthly) or disaggregates (daily). Pentadal CHIRP values are disaggregated to daily precipitation estimates based on daily CFS fields rescaled to 0.05° resolution. At each pixel, the CHIRP pentad total is redistributed in proportion to the daily values of the CFS.

The CHIRP relies on two global geosynchronous TIR archives, the 1981–2008 Globally Gridded Satellite (GriSat) archive produced by NOAA’s National Climate Data Center^27^ and the 2000-present NOAA Climate Prediction Center dataset^21^ (CPC TIR). The first few years of the GriSat archive frequently have pixels with missing TIR observations. When these missing TIR values result in missing CHIRP estimates, the missing CHIRP values are filled using unbiased (% anomaly * CHPclim) CFS version 2 reanalysis fields^17^. The CPC TIR data are used from 2000-present. A 2000–2008 overlap between the GriSat and CPC TIR data are used to estimate a single offset adjustment for the GriSat data for each grid cell and month. GriSat and CPC TIR precipitation values were found to have little systematic difference during the period of overlap.

### Station data merging

The CHIRPS station processing stream incorporates data from five public data streams and several private archives. The public data streams are the GHCN monthly, GHCN daily, Global Summary of the Day (GSOD), GTS and Southern African Science Service Centre for Climate Change and Adaptive Land Management (SASSCAL, www.sasscalweathernet.org). GTS data are collected daily; GHCN, GSOD and SASSCAL data are updated monthly. Additional observations have been provided by national meteorological agencies, primarily in Mexico, Central America, South America, and sub-Saharan Africa. Currently, the Climate Hazards Group (CHG) archive has almost 600 million daily observations since 1981 and another 600 million before that year from over 200,000 locations. The typical number of observations going into the monthly CHIRPS ranges from greater than 32,000 in the early 1980s to less than 14,000 in 2014, ftp://ftp.chg.ucsb.edu/pub/org/chg/products/CHIRPS-2.0/diagnostics/stations-perMonth-byRegion/pngs/all.station.count.CHIRPS-v2.0.png. Focusing on all countries except Australia, Brazil, Canada, Colombia, Mexico, and the USA (which provide the largest number of observations), we find a more concerning decline in which the number of observations drops from over 7,000 in 1981 to less than 4,000 after 2010, ftp://ftp.chg.ucsb.edu/pub/org/chg/products/CHIRPS-2.0/diagnostics/stations-perMonth-byRegion/pngs/Global-top6.station.count.CHIRPS-v2.0.png. In Africa (excluding the Republic of South Africa), we find about 2,400 stations in the early 1980s, and only about 500 after 2010, ftp://ftp.chg.ucsb.edu/pub/org/chg/products/CHIRPS-2.0/diagnostics/stations-perMonth-byRegion/pngs/Africa.station.count.CHIRPS-v2.0.png. A similar decline can be observed in central southwest Asia (ftp://ftp.chg.ucsb.edu/pub/org/chg/products/CHIRPS-2.0/diagnostics/stations-perMonth-byRegion/pngs/Central_Middle_Asia.station.count.CHIRPS-v2.0.png) and South America (ftp://ftp.chg.ucsb.edu/pub/org/chg/products/CHIRPS-2.0/diagnostics/stations-perMonth-byRegion/pngs/South_America.station.count.CHIRPS-v2.0.png).

The CHG station archive is used to define a set of global anchor station locations (*N*=47,390). This system produces more robust long-term time series with fewer stations that blink in and out. N-station time series of monthly and pentadal precipitation are created using a ranked list of preferential sources, with national meteorological archives typically ranking first and GSOD and GTS listing last. This ordering was selected because the automated GSOD and GTS systems are known to exhibit problems associated with missing data marked as zero and erroneously repeated daily observations. The highest quality data sources are used as the initial set of anchor stations, and then to increase spatial coverage, stations from other sources are added to the set in order of source ranking. A station is added to the set if there is not already an anchor station within 5 km. If there is already an anchor station within this distance the station is flagged to be used only for filling in missing data at the anchor station location. Automatic quality control routines screen for extreme values based on standardized anomalies (>|±4σ|), very large absolute values, and large ratios (>5×) of the CHIRP. These extreme values are not used in the CHIRPS blending process. In some countries a large number of missing values in the daily GTS and GSOD are coded as zeros. To reduce (but not eliminate) these false zeros, a screening process was developed that: i) disaggregates the global CHIRP pentads to days using the CFS, ii) and screens out data where the GTS or GSOD is zero and the daily CHIRP is above normal.

### CHIRPS

**Figure 2:**
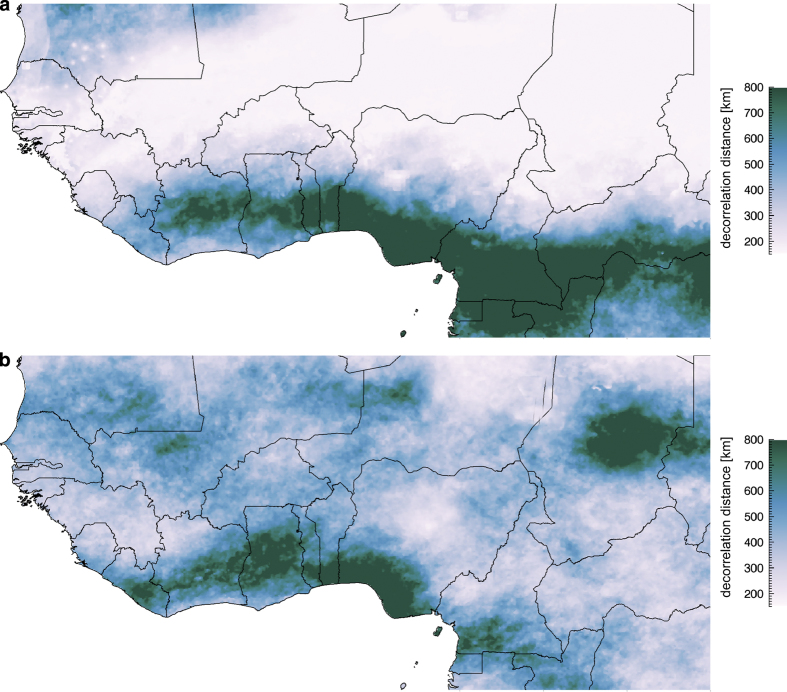
Maps of CHIRPS decorrelation distances. (**a**) February. (**b**) August decorrelation distances.

The CHIRPS station blending procedure is a modified inverse distance weighting algorithm that has several unique characteristics. The first of these is the use of the CHIRP to define a local decorrelation distance; this distance is where the estimated point-to-point correlation is zero. Fig. 2 provides examples of the decorrelation distance for February and August in West Africa. To generate these values, time series of CHIRP data were used to calculate the average correlation at a distance of 1.5° for each grid cell. This correlation, and the assumption that the expected correlation is 1 when distance is 0 allows for estimation of a decorrelation slope, which is used to estimate the zero-correlation distance. The correlation structure evolves in space and time, tending to be stronger in areas of heavy well-organized convection.

For any given pixel, the CHIRPS blending procedure is based on a weighted average of the ratios between the five closest stations and the CHIRP: **b**_1..5_=(**s**_1..5_+ε)/(**c**_1..5_+ε), where **b** is a 5-element vector of bias ratios, **s** is a 5-element vector of station observations, **c** is a 5-element vector of CHIRP values. A small epsilon number (ε) is included in the denominator and numerator to handle zero or near zero CHIRP values. Ratios greater than three are capped at three. Bias values (**b**) for any station beyond the decorrelation distance are assumed to be 1.

A weighted average of these five bias values is then calculated, based on their distances and the decorrelation slope described above. A further calculation adjusts this value based on the expected correlation with the nearest station (R_ns_) and the expected correlation between ‘true’ precipitation values and the CHIRP data (R_CHIRP_). R_CHIRP_ is set a priori at 0.5 based on validation results. These correlations are used to assign a weighting value for the CHIRP: α=R_CHIRP_/(R_CHIRP_+R_ns_). The final CHIRPS estimate is a combination of unadjusted and bias-adjusted CHIRP data: CHIRPS=αCHIRP+(1-α)bCHIRP. Thus, even in the presence of co-located stations, the CHIRPS will have some influence from the CHIRP.

### Code availability

The CHIRPS data products are derived using approximately 12,000 lines of code written in the Interactive Data Language. While not written as a portable library or toolset, access to the code is not restricted, and it is available upon request.

## Data Records

### CHIRP and CHIRPS processing schedules

CHIRPS data are available on our FTP site (ftp://ftp.chg.ucsb.edu/pub/org/chg/products/CHIRPS-2.0/) for a number of time steps, spatial domains, and formats from 1981 to present. A preliminary product is produced every pentad with a 2-day lag using GTS and Mexico data only, are also available on these days. At the end of the month, a monthly preliminary CHIRPS is created as the sum of the six preliminary pentads.

During the 3rd week of the month, when nearly all of the previous month’s GHCN and GSOD data has become available, the final CHIRPS pentads and monthly files are created. Final pentad and monthly station data files are created and quality controlled. These station datasets are combined with monthly and pentadal CHIRP grids as described above. The six final pentad files are adjusted to ensure that their totals equal the final monthly CHIRPS values.

Daily CHIRPS are then produced for the globe by using daily CCD data to identify non-precipitating days. Whenever the daily CCD is zero, precipitation is assumed to be zero. Missing CCD days are assumed to be non-precipitating days if the CFS precipitation is less than 3.5 mm per day. This threshold was determined through comparisons with the Sheffield dataset^28^. Daily CCD values are translated to precipitation using the regression estimates described in our Methods section. Each pentad’s total precipitation is then allocated proportionally among the days with precipitation. Where this fails to distribute the monthly precipitation, because of warm rain events missed by daily CCD estimates, an extra step is applied using CFS daily values and the expected number of raindays for that pixel to downscale the monthly value to daily. Six-hourly disaggregations are also produced by using the ratios between the daily and six-hourly CFS data.

### CHIRP and CHIRPS data formats

CHIRPS data are available on our FTP site (ftp://ftp.chg.ucsb.edu/pub/org/chg/products/CHIRPS-2.0/) for a number of time steps, spatial domains, and formats from 1981 to present. A preliminary product is produced every pentad with a 2-day lag using GTS and Mexico data only. The final CHIRPS product is produced once a month with an approximate 3-week lag using all available station data.

CHIRPS data is available from 6-hourly to 3-monthly aggregates. Almost all data has an 0.05°×0.05° degree spatial resolution. Some of the Africa daily data is reduced to 0.25°×0.25° spatial resolution to support land surface modeling activities. The three main spatial domains are Global (7200×2000 pixels, 180°W to 180°E, 50°N to 50°S), Africa (1500×1600 pixels, 20°W to 55°E, 40°N to 40°S) and Central America-Caribbean (720×350 pixels, 93°W to 57°W, 23.5°N to 6°N).

CHIRPS data are provided in NetCDF, GeoTiff, and Esri BIL formats. The units are mm per time period, e.g., mm per day, mm per pentad, mm per month. Supporting data are created each month to compliment the CHIRPS data including: i) density of stations used, ii) browse images of Africa for several time steps, iii) lists of locations and names of all stations used for each month, and iv) the number of stations used per month for each country. Please see links at ftp://ftp.chg.ucsb.edu/pub/org/chg/products/CHIRPS-2.0 and http://chg.ucsb.edu/data/chirps/. The data reviewed for this paper extend from January 1981 to August 2015. The CHIRPS v2.0 dataset will continue to grow over time as new TIR and station data become available.

## Technical Validation

### CHPclim validation results

Table 1 presents CHPclim validation results for Afghanistan, Colombia, Ethiopia, Mexico and the Sahel (Senegal, Burkina Faso, Mali, Niger and Chad), based on the more extensive study presented in our paper describing the CHPclim^19^. In each case, additional high-quality gauge data were obtained from national meteorological agencies. These data were screened, and only values not in the FAO or GHCN archives were retained for the validation study. For each validation station, the CHPclim, CRU, or WorldClim grid cell containing the station was extracted. Monthly validation statistics were calculated, and then averaged across all 12 months. While all three climatologies capture well the overall mean in Colombia, Ethiopia, and Mexico, Afghanistan and the Sahel, the CRU and WorldClim exhibit large (>±15%) biases. This may be due to poor performance by the thin plate spline fits in these data sparse regions. The CHPclim bias in these regions is low, ~3% or less. In Colombia, Afghanistan, Ethiopia and the Sahel the CHPclim Mean Absolute Error (MAE) values are about half as large as those found in the CRU climatology. The variance explained is also substantially larger. While the WorldClim performs well in Colombia, in the Sahel, Afghanistan, and Ethiopia the CHPclim performed better. CHPclim appears to perform well in data-sparse regions with complex terrain.

### CHIRP and CHIRPS wet season validation results

Because we are primarily interested in agricultural drought monitoring, we focus here on precipitation estimation performance during the wettest three months of the year (Fig. 1b). This allows us to compactly summarize error statistics in a single map. Using GPCC precipitation as our baseline, we present comparisons with the CHIRP and CHIRPS, TMPA 3B42 v7 real-time (RT) estimates with no gauge data, the CFS and ECMWF reanalysis products, and the CPC Unified interpolated gauge products. These products were chosen because they have coverage and latencies comparable to the CHIRP and CHIRPS. The CPC Unified product is based on interpolated daily precipitation^29^. A common 2000–2010 time period was used in our analysis. Areas with mean wet season precipitation totals of less than 50 mm were masked.

**Figure 3:**
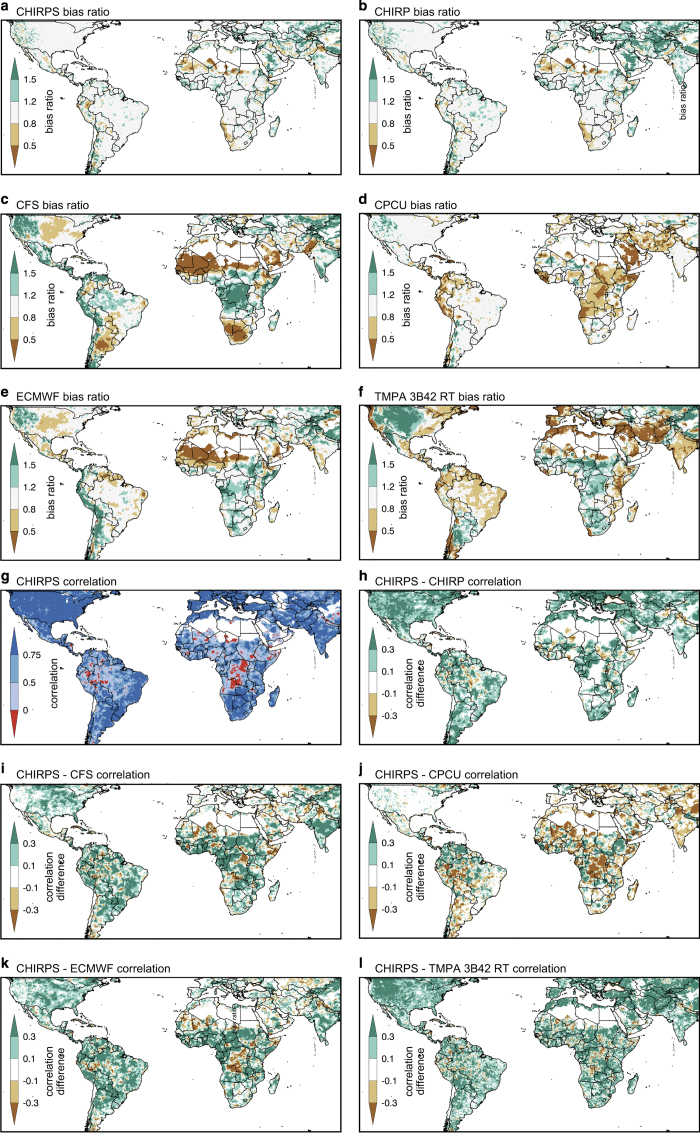
Wet season validation results. (**a**–**f**) Wet season bias based on comparison with 2000–2010 GPCC data. Bias is defined as the ratio between the mean of the validated product and mean GPCC precipitation. (**g**) Wet season correlations between the CHIRPS and GPCC (**h**–**l**). Difference between the CHIRPS and GPCC correlation map (**g**) and the corresponding correlation maps with the other products and the GPCC.

Figure 3a–f shows wet season bias estimates. The CHIRPS, and to a lesser degree the CHIRP, clearly exhibit less bias than the other products. This low bias arises from the use of the CHPclim, and the inclusion of station data in the CHIRPS. Note that similar station data are used in the GPCC and the CHIRPS. The CHIRP, however, does not benefit from these observations, and the CHIRP bias is substantially better than the TMPA 3B42 RT, CFS, and ECMWF (Fig. 3; Table 2). Even the CPC Unified, which is based on station data, substantially underestimates precipitation in East Africa, the Middle East, and southwest Asia. This is probably due to low station densities in those areas. The CHIRP and CHIRPS mean percent biases (Table 2) over semi-global domain in Fig. 3 (126°W-90°E), Africa, and the USA are similar to those found in the CPC Unified and substantially lower than those found for the CFS, ECMWF, and TMPA 3B42 RT7 products.

We next examine wet season correlations between the GPCC and other precipitation estimates (Fig. 3g–l). Figure 3g shows the 2000–2010 correlation between the CHIRPS and GPCC; in many regions a good (R>0.75) correspondence is found. These areas tend to be fairly well monitored by in situ observations (CHIRPS diagnostics are available at http://chg.ucsb.edu/data/chirps/#_Diagnostics). Some areas with very low or negative correlations, like the Democratic Republic of Congo (DRC), report virtually no rain gauge information, ftp://ftp.chg.ucsb.edu/pub/org/chg/products/CHIRPS-2.0/diagnostics/stations-perMonth-byCountry/pngs/Drc.049.station.count.CHIRPS-v2.0.png. Differencing the GPCC/CHIRPS correlations with the GPCC/other product correlation fields (Fig. 3h–l) highlights their relative performance. Green (brown) locations identify areas with higher (lower) correlations with GPCC, compared to the GPCC/CHIRPS correlation map. As expected, CHIRPS almost always exhibits higher correlations than the non-station based estimates: CHIRP, CFS, ECMWF and TMPA 3B42 RT. The two satellite-only products (the CHIRP and TMPA 3B42 RT7) exhibit similar performance (Fig. 3h,l; Table 2). The overall performance of these satellite-only estimates (the CHIRP and TMPA 3B42 RT7) appears worse than the ECMWF and CFS correlation performance (Fig. 3i,k; Table 2). The EMCWF and CFS correlation performance appears substantially worse than the CHIRPS but better than the CHIRP and TMPA 3B42 RT7 over North America, Europe and parts of Asia; presumably because of their improved representation of precipitation processes and storm systems in these areas (e.g., cold season frontal storm systems as opposed to deep convective tropical systems). Given these promising correlations, it may be possible to produce future versions of CHIRPS that incorporate these model precipitation estimates as well. CPC Unified (Fig. 3j) exhibits better correlations than the CHIRPS in a few places like the DRC and Bangladesh—these locations tend to have very few station observations. Since about 1997, the number of available stations from the DRC, a country about four times the size of France, has been essentially zero.

Mean absolute error (MAE) analysis^30^ provides a simultaneous evaluation of systematic error (bias) and random errors (which are inversely related to correlations). Table 2 summarizes MAE statistics (mm over the 3-month wet seasons shown in Fig. 1b) for the semi-global domain in Fig. 3 (126°W-90°E), Africa, and the USA. Under these criteria, the low bias of CHIRPS translates into the lowest MAE scores. Even without station inputs, the MAE performance of the CHIRP is similar over Africa and the semi-globe to the CPC Unified, and substantially lower than the CFS, ECMWF, and TMPA 3B42 RT7.

### Validation results for Colombia

**Figure 4:**
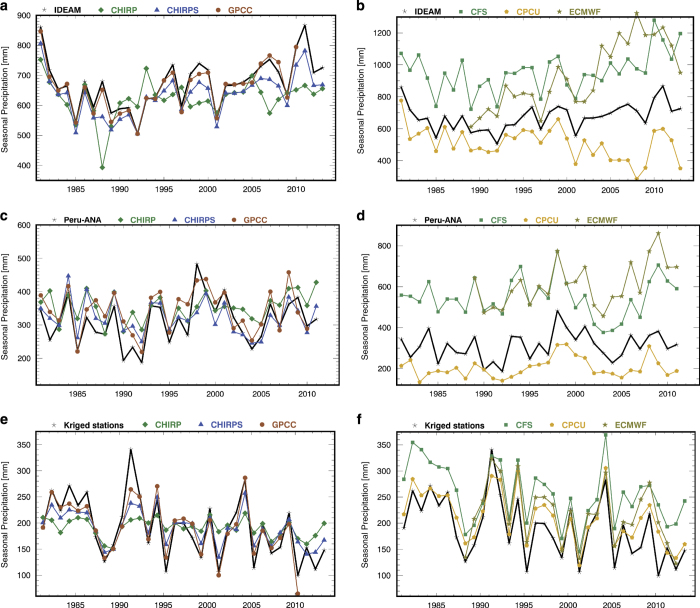
Validation time series. Results for Colombia (**a**,**b**), Peru (**c**,**d**), and SWNA (**e**,**f**).

We next present a brief validation study based on 10% (338) of the total 3,380 stations from Colombia, obtained from the Instituto de Hidrología, Meteorología y Estudios Ambientales (IDEAM). This example was chosen because the collaboration with the meteorological department (IDEAM) provided a unique setting to test the CHIRPS in an area of complex terrain dominated by tropical warm rain processes. While these stations were included in the final CHIRPS release, they were first withheld, and monthly ‘final’ CHIRPS values were estimated. The monthly CHIRPS, CHIRP, CFS, CPC Unified, ECMWF, and GPCC values at the station locations were extracted. The mean of these locations (for each month) was calculated and then summed across the main rainy season (September-November) for each year. Figure 4a shows the observed IDEAM time series and the CHIRP, CHIRPS, and GPCC estimates. The GPCC tracks extremely well with the IDEAM dataset (R=0.96, MAE 21 mm), but ends in 2010 (Version 7 of the GPCC now goes to 2013). The CHIRPS performance is slightly worse (R=0.97, MAE 38 mm), but an advantage of CHIRPS is that new estimates are available with relatively low latencies.

Unfortunately, the CCD-based CHIRP provides little information on year-to-year September-November rainfall variations (R=0.38). The disappointing performance of the CHIRP, however, should be contrasted with the apparent non-stationarity of the ECMWF, CFS, and CPC Unified time series (Fig. 4b). The model-based CFS and ECMWF overestimate precipitation substantially, and show very large recent precipitation increases. These time series do, however, have reasonably high interannual correlations (0.76 for CFS and 0.72 for ECMWF). The CPC Unified correlation is low (0.45) and exhibits a substantial decline that may be related to changes in the observation network. The MAE values for the CPC Unified, CFS, and ECMWF datasets were very high (154, 221, and 213 mm).

### Validation results for Peru

We next present another validation study based on 403 stations from Peru’s main rainy season (January-March). These data were provided by Peru’s National Water Authority. While these data were included in CHIRPS, they were first withheld to allow a validation study based on the same compositing process as described above. Once again (Fig. 4c), the CHIRPS performance (R=0.72, MAE 37 mm) was found to be comparable to that of the GPCC (R=0.82, MAE 42 mm). For this region and season, the CHIRP performance was moderately good (R=0.6, MAE 54 mm). Once again (Fig. 4d), we find that the model-based estimates correlate well (R=0.71 for CFS, R=0.76 for ECMWF), but have dramatic tendencies to overestimate that result in high MAE values (235 and 296 mm, respectively). The correlation of the CPC Unified dataset (R=0.6) was similar to CHIRP, with a substantial underestimation problem and high MAE (108 mm).

### Validation results for southwestern North America

We next present a brief validation for southwestern North America (SWNA, 118°E-102°E, 24°N-43°N) for October-June precipitation. The ‘truth’ data used in this example are a dense set of GHCN station data block kriged for the region of interest. The CHIRP appears to substantially underestimate the variance of SWNA precipitation, but does exhibit a reasonable correlation (0.70). The CFS and ECMWF tend to overestimate, but have high correlation values (0.85 and 0.87, respectively), on par with the correlations of the CPC Unified, GPCC, and CHIRPS (0.90, 0.93, and 0.91, respectively). The CHIRPS, which is essentially a blend of the CHIRP and the GPCC-like interpolated station data, ‘inherits’ some of the low variance of the CHIRP, and therefore tends to underestimate the full swings between the high and lows shown in the kriged time series present in Fig. 4e.

### Summary of validation results

The validation results presented here illustrate some of the advantages and weaknesses of the different products examined. Wet season precipitation estimates from climate models tended to have reasonably high correlations with observed data (e.g., in the Middle East, North Africa, North America, Europe and Asia (Fig. 3i–k)) but to also have important bias issues (e.g., Columbia and Peru (Fig. 4a–d)). For remote drought monitoring applications, the large biases and potential non-stationarities of model estimates could pose serious obstacles to effective monitoring and environmental change detection. For example, the Colombia ECMWF precipitation data shown in Fig. 4b increases from about 800 mm between 1997–2004 to near 1200 mm between 2007 and 2014. This spurious ‘environmental change’ completely obscures the 2009 Colombian drought. In areas like southwestern North America, where radiosonde networks are dense, the CFS and ECMWF models provide accurate and independent sources of precipitation information.

While the CPC-Unified also performs well in the SWNA, where gauge density is relatively high, we find that in Colombia (Fig. 4b) its performance degrades substantially after 2002, with rainfall decreasing in contrast to the observed increases. The CPC Unified dataset, which is based on daily interpolated station data^29^, appears susceptible to spurious ‘excursions’, which are probably related to changes in the underlying station network. In some areas CPC-Unified exhibited a higher correlation with GPCC than did CHIRPS. This occurred in parts of Africa, Asia, and South America where correlations were still low. Higher numbers of reporting gauges in these areas would improve both products.

The CHIRPS dataset appears well suited to its original objective—to provide GPCC-like performance with lower latencies. The CHIRPS bias is low (Fig. 4), as was intended in its design, and independent validations in Peru and Colombia indicate success on this aspect of verisimilitude and GPCC-like performance. CHIRPS correlations with wet season GPCC precipitation were greater than R>0.75 in many areas of the world and substantially outperformed the climate model estimates in South America, Africa and India. Correlations were higher than TMPA 3B42 RT in most regions. While more analysis of CHIRPS performance, especially at sub-seasonal scales, will be required in the future, our preliminary global and national validation studies indicate promising results.

## Usage Notes

In this section, we present a sample application of the CHIRPS, based on hydrologic simulations over East Africa, which has experienced exceptional drying during boreal spring^31^. The reasonably long period of record, low latency, high resolution and daily disaggregations of the CHIRPS make it suitable for hydrologic modeling. Focusing on East Africa, and especially southeastern (SE) Ethiopia, we demonstrate how CHIRPS can be used to support humanitarian relief efforts while guiding climate-smart development. In eastern East Africa, the interaction of declining boreal spring rains, population growth, cropland extensification, and land cover change and degradation may be enhancing food insecurity^30–33^. The CHIRPS dataset has been used to explore the Eastern Africa nexus of climate change, population growth, and vegetation declines^34^ and identify links between drought and low birth weights^35^. In this sample application of the CHIRPS, we i) use the dataset to drive a hydrologic model, the Variable Infiltration Capacity (VIC) model^36,37^, ii) analyze recent (post-1999) changes in soil moisture (SM), evapotranspiration (ET), rainfall, and air temperatures, and iii) consider how the near real-time CHIRPS can be used with sea surface temperature (SST) conditions to predict most of the recent severe droughts in southeastern Ethiopia.

High-quality, temporally consistent and near real-time precipitation datasets such as CHIRPS can help identify environmental changes, quantify the important role played by warming air temperatures and play an important role in seasonal drought prediction^38–42^. CHIRPS can be used to force hydrologic models and simulate near-real time initial hydrologic conditions. State of soil moisture, groundwater and snow pack^43^ have been shown to contribute to drought prediction skill^44^, which is valuable given that there is limited skill in climate forecasts (mainly for precipitation)^41,42^ beyond approximately 2 months. Furthermore, statistical models for drought prediction have also been shown to be sensitive to and derive their skill from the initial soil moisture state^45,46^. These type of models can predict extremes like the 2011 East Africa drought. Below we show that the near real-time CHIRPS can help capture this potentially valuable hydrologic information and support enhanced drought early warning.

### Context—southeast Ethiopia

**Figure 5:**
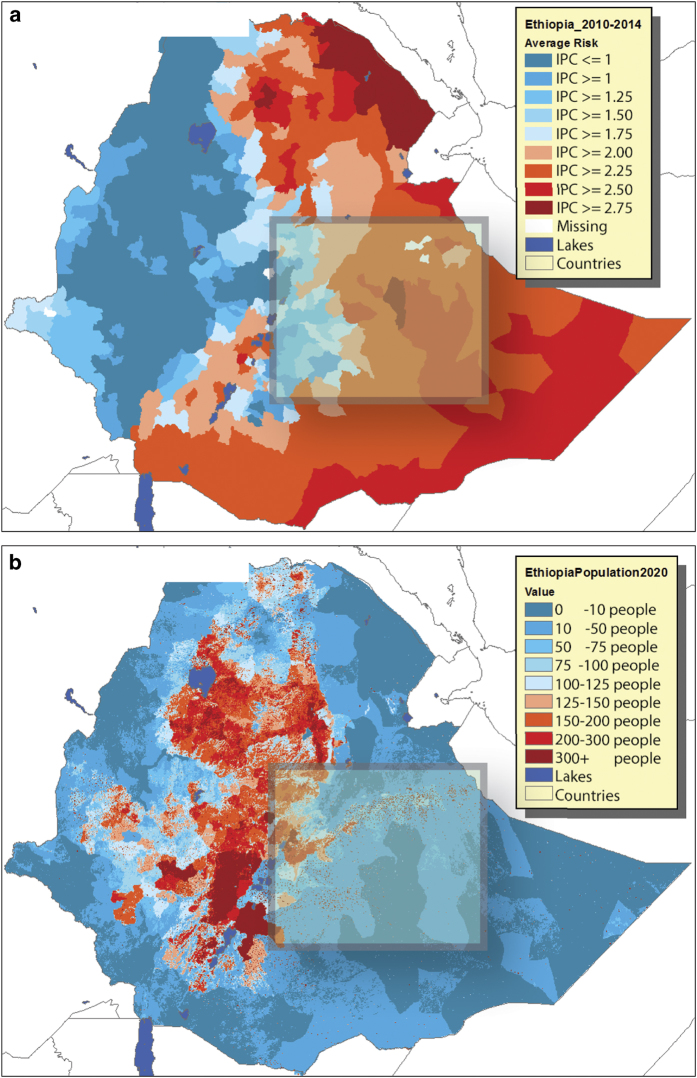
Ethiopia food insecurity and population. (**a**) Average 2010–2014 FEWS NET food insecurity status (Integrated Phase Classification) for Ethiopia. (**b**) 2020 Gridded Population of the World population estimates for Ethiopia, expressed as people per 2.5 arc-minute grid cell.

**Figure 6:**
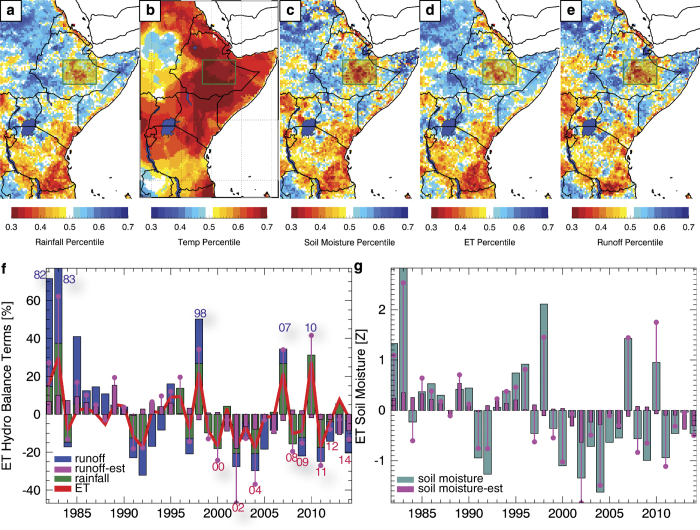
Ethiopia hydrologic simulation results. (**a**) Average 1999–2014 October-September CHIRPS rainfall percentiles, based on a 1981–2014 baseline period. (**b**) Same for GISS air temperature anomalies. (**c**–**e**) Same, but for VIC hydrologic model simulations. (**f**) 1981–2014 VIC runoff (blue bars), VIC evapotranspiration (red line) and CHIRPS rainfall (green bars). Purple dots show regression estimates of runoff based on annual rainfall and average air temperatures (cross-validated R^2^=0.63). Purple bars show estimates based solely on air temperature variations. Anomalous pluvials and droughts are indicated with years. (**g**) Standardized VIC soil moisture anomalies. Purple dots show regression estimates based on annual rainfall and temperatures (cross-validated R^2^=0.88); purple bars show estimates based solely on air temperature variations.

While the VIC simulations were run across the Greater Horn of Africa, our analysis focuses on southeastern Ethiopia (38.5°E-44°E, 6°N-10°N) because this is a region with chronic food insecurity (Fig. 5a) and with a large population (Fig. 5b) that is growing rapidly^34^. The region exhibits declining precipitation, ET, SM, and runoff as seen in the data (Fig. 6a). Between 2010 and 2014, much of this region was classified by FEWS NET as facing food stress or crisis conditions, on average, based on Integrated Food Security Phase Classification (IPC) assessments (www.fews.net) (Fig. 5a). IPC values ranging between 2 and 3 indicate recurrent food crises associated with chronic malnutrition rates of 10–15%, acute dietary diversity deficits, and accelerated and critical depletion of livelihood assets; an IPC value of 3 or more denotes a humanitarian emergency—with acute malnutrition affecting more than 15% of the population, severe food access shortages, and near complete and irreversible depletion or loss of livelihood assets (IPC, 2008). Gridded Population of the World estimates for 2020 for this region are 32,630,400, a value that is expected to almost double by 2050 to 62,000,928 (ref. 47). Boreal spring rains in this region have exhibited substantial declines since the 1980s (refs 31,32). Given that the persistence of soil moisture conditions often presents opportunities for effective hydrologic forecasts^43^, we examine here whether CHIRPS can help provide effective soil moisture predictions for annual southeastern Ethiopia soil moisture. We also explore the relative contributions of rainfall declines and warming air temperatures.

### Hydrologic simulation percentiles for 1999–2104

In this section we examine 1981–2014 hydrologic changes, based on annual October-September accumulations of runoff, ET, and 0 to 40 cm soil moisture from VIC model simulations (the top two VIC soil moisture layers). The VIC model^36,37^ has previously been used to analyze recent East African droughts^43,48,49^. The VIC model was run in the water balance mode using daily CHIRPS, temperature maxima and minima and wind speed. Daily temperature and wind speed data used to run model simulation from 1981–2010 were the same as those used by Princeton African Drought Monitor^49^. Post-2010 daily temperature data were generated by constraining the Global Ensemble Forecast System (GEFS) daily temperature minimum and maximum analysis fields with gridded monthly observations of minimum and maximum air temperatures produced by CRU^9^. Regressions between monthly 1901–2013 CRU temperature fields and Goddard Institute for Space Science (GISS) gridded air temperature anomalies^50^ were used with 2014 GISS anomalies to constrain the 2014 GEFS temperature anomalies. Wind speed data post-2010 were simply the daily climatological mean for the period 1981–2010.

In Fig. 6a,b we show the average of the annual (October-September) 1999–2014 CHIRPS precipitation and GISS air temperature values, respectively, expressed as expressed as percentiles. These maps were generated by i) calculating the average 1999–2014 rank at each grid cell based on the full 1981–2014 time series and ii) dividing this average rank by 33, the number of water years. An October-September water year was chosen to highlight the region's sensitivity to back-to-back Indo-Pacific-forced boreal fall and spring droughts^51^. While recent drying trends have mostly arisen during spring, the worst recent food security crises in East Africa (like that occurring in 2011) have typically been associated with multi-season dry spells. We focus, therefore, on annual soil moisture and runoff extremes, and our capability to predict southeastern Ethiopia droughts at the beginning of the main April-September rains. We refer to the 1981–1982 October-September water year as ‘1982’. Since 1999, parts of eastern East Africa have experienced below normal rainfall (yellow and red areas in Fig. 6a). Air temperatures have also increased (Fig. 6b), especially over northeastern Kenya and Ethiopia. Spatial patterns of the VIC soil moisture, ET, and runoff 1999–2014 percentile values (Fig. 6c–e) follow closely the CHIRPS precipitation anomalies (Fig. 6a); we will show below, however, that warm temperatures have also contributed substantially to increased aridity in Ethiopia.

Focusing on our southeastern Ethiopia domain, we present time series of precipitation, ET, and runoff, expressed as percent anomalies of the 1982–2014 mean (Fig. 6f). These percentiles are calculated with regard to the each grid cell’s 33-year history. In general, the coefficient of variation of the runoff is much higher than that of the rainfall or ET time series. Since 1999, only 2 years have exhibited above normal ET, runoff, or rainfall. This increased frequency of droughts^52^ is probably due to tropical Pacific SST forcing^53^. The most recent FEWS NET research links these droughts to an exceptionally strong tropical Pacific SST gradient^32,43,48,54,55^, caused largely by anthropogenic warming of the western Pacific^56^ and a natural decadal cooling of the eastern Pacific^57^.

We can examine the relative contributions of annual rainfall and temperature to annual southeastern Ethiopia runoff using leave-one-out cross-validated (CV) regressions (CV R^2^=0.63). Figure 6f shows the corresponding runoff estimates based on i) precipitation and GISS temperatures, and ii) GISS temperatures alone. The southeastern Ethiopia VIC runoff declined by ~20 percent between 1981–1998 and 1999–2014. Regression estimates suggest that about half (10 percent) of this decline may be attributed to lower rainfall and that the other half may be attributed to warming air temperatures.

We can apply a similar analysis to southeastern Ethiopia soil moisture anomalies (Fig. 6g). We have expressed the soil moisture time series using standardized anomalies to facilitate their interpretation in a drought monitoring context. Since 1999, only 2 years have exhibited above normal soil moisture, and the change between the 1981–1998 and 1999–2014 standardized anomalies was −0.9. A cross-validated regression predicting annual soil moisture based on rainfall and air temperatures fits almost perfectly (CV R^2^ 0.88). These estimates (Fig. 6g), suggested that most (70%) of the decline in soil moisture was caused by reductions in precipitation, while air temperature increases may have accounted for about 30% of the decline.

The result that temperature change potentially accounted for 50 and 30% of runoff and soil moisture declines, respectively, may have important implications for future impacts of climate change. These estimated temperature influences are shown with purple bars on Fig. 6f,g. Based on the runoff results, warming might have prevented any change in runoff even if the rainfall trend had been in the opposite direction (a wetting trend). Note that such results can be highly model sensitive^58^, and these findings will need to be verified with more models. We plan future analyses using multiple hydrologic models in conjunction with numerical experiments, isolating precipitation and temperatures impacts through multiple suites of simulations.

### Predictions of southeastern Ethiopia hydrologic extremes

Can CHIRPS help us predict southeastern Ethiopia droughts? As context, consider early April of 2011. The fall 2010 rains had been poor across the Horn of Africa, and millions of pastoralists and farmers faced severe food shortages unless conditions improved. What was the chance that drought would persist? Datasets like CHIRPS can help us answer this question by providing a basis for hydrologic simulations in near real-time and by giving us a firm foundation for exploring teleconnections and prediction. This requires precipitation estimates with both reasonably long periods of record and reasonably low latencies. Present soil conditions are often a good indicator of future hydrologic outcomes. Average October-March southeastern Ethiopia precipitation comprises on the order of 25% of the annual total (145 mm out of 622 mm). The main rains for this region come between April and September. However, October-March southeastern Ethiopia soil moisture conditions are a good indicator of the overall performance of the mean October-September soil moisture conditions (CV R^2^ 0.47). While persistence of soil moisture anomalies certainly accounts for some of this predictability^43^, we find a similar persistence between October-March and April-September CHIRPS rainfall anomalies as well (CV R^2^ 0.46), perhaps because both the October-March and April-September rainy seasons are suppressed by warm SSTs in the Indo-Pacific warm pool and stronger west to east tropical Pacific SST gradients^54,59^. This persistence between seasons has also been noted in another recent study^60^. We show how climate and land surface persistence can be combined to make effective hydrologic forecasts at a coarse spatial and temporal scale for southeastern Ethiopia.

**Figure 7:**
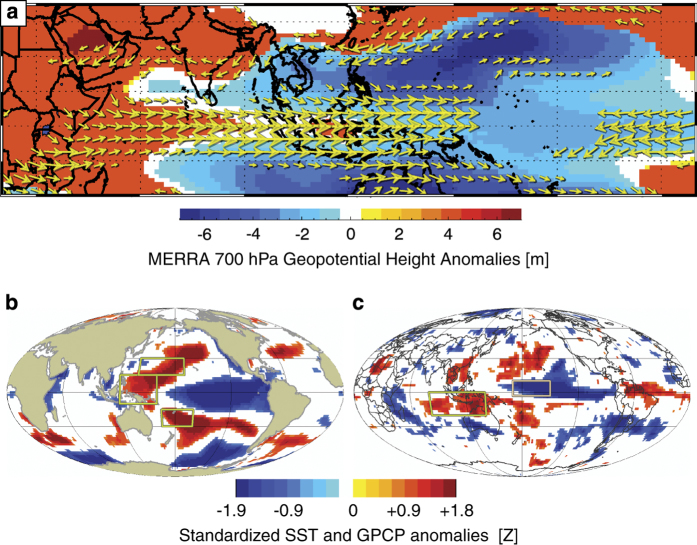
Climate composites (droughts minus pluvials) for the anomalous years noted in Fig. 6f. (**a**) October-September MERRA 700 hPa geopotential heights and winds. (**b**) Standardized January-March NOAA Extended Reconstructed SSTs. (**c**) Standardized January-March GPCP precipitation. All composites screened for significance at *P*=0.1.

Figure 7 shows January-March composites of the large-scale circulation based on the difference between the dry and wet years noted above. The January-March time period shows climate conditions just as rains commence in earnest over southeastern Ethiopia. The purpose of this plot is to show that just before the onset of the rains, the large-scale Indo-Pacific climate system exhibits large coherent anomalies consistent with an intensification of the Walker circulation. Conditions like these can be used by East African climate experts to predict some boreal spring droughts^32,43,61^. Coupled and uncoupled climate model simulations indicate that when there is a strong tropical SST gradient during boreal winter, with warm SST in the western Pacific and cool SST in the central Pacific, predictable rainfall deficits often follow during East Africa boreal spring rains.

In Fig. 7a we see a large difference between the Indo-Pacific climate system in the months preceding the driest and wettest years in southeastern Ethiopia. These years are indicated in Fig. 6f. Using 700 hPa winds and geopotential height anomalies from the Modern-Era Retrospective Analysis for Research and Applications (MERRA) reanalysis^62^, we can see that the difference between dry and wet years is characterized by strong anomalous westerly low winds extending from East Africa to the maritime continent. These anomalous winds are forced by a stronger equatorial height gradient between East Africa and the eastern Indian Ocean, and cells of sub-tropical low pressure to the north and south of the maritime continent. This type of circulation change probably reduces moisture transports into Ethiopia during both boreal spring^63^ and summer^64^, and appears related to warmer West Pacific SSTs^51^. While Ethiopia does not appear to be experiencing precipitation declines at a national scale^65^, the western part of country has been getting wetter while the eastern part has seen rainfall decreases^66^.

The SST^67^ and GPCP^68^ precipitation responses shown in Fig. 7b,c are characteristic of conditions associated with recent East Africa precipitation declines^52,53^ and drought predictions^32,43,48,55^. Warming in the western Pacific and cooling in the eastern Pacific intensify the climatological SST gradient supporting an intensification of the Walker Circulation with more (less) precipitation over the western (central) Pacific (Fig. 7c). The diabatic forcing from the increased Indo-Pacific precipitation helps force an equatorial Rossby wave response over the Indian Ocean^54,69,70^, producing westerly wind anomalies (Fig. 7a) and drying over eastern East Africa (Fig. 7c). Presumably, the January-March GPCP rainfall deficits across Tanzania, Kenya, and southern Somalia in Fig. 7c presage hotter drier conditions in southeastern Ethiopia in April-September.

Western Pacific and central Pacific SSTs exhibit substantial persistence, and partial correlations, controlling for antecedent October-March soil moisture conditions, show that January-March SST averaged over the ‘western V’^56^ boxes shown in Fig. 7b are reasonably well correlated with April-September southeastern Ethiopia soil moisture conditions (R=0.5). Exceptionally warm SSTs in this region have contributed to the 2014 East African spring drought^32,48^ and the long term decline in rainfall observed in the Centennial Trends precipitation product^31^.

**Figure 8:**
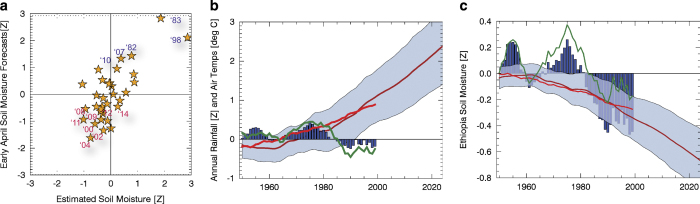
Estimates of Ethiopian soil moisture. (**a**) Cross-validated forecasts of October-September SE Ethiopia soil moisture based on observed October-March soil moisture and observed January-March West Pacific SSTs. (**b**) 15-yr averages of SE Ethiopian October-September precipitation (blue bars), March-June rainfall (green line), GISS air temperatures (red line) and CMIP5 ensemble mean air temperature value (dark red line) with 95% confidence intervals. (**c**) Regression estimates of SE Ethiopian 15-yr averages of soil moisture based on observed rainfall and air temperatures from Fig. 8b (blue bars), based on GISS air temperatures only (red line) and on CenTrends rainfall only (green line). Also shown are the same regression estimates based on the CMIP5 ensemble average (dark red line) and 95% confidence interval spread.

Combining these January-March western V SSTs with the observed October-March southeastern Ethiopia soil moisture anomalies supports effective forecasts of most of this region’s hydrologic extremes (Fig. 8a). The overall cross-validated correlation of this simple forecast model is 0.78. Most (7 out of 8) of the targeted dry anomalies were predicted to be below normal, and most (4 out of 5) of the wet anomalies were predicted reasonably well. Thus, while southeastern Ethiopia has been experiencing declines in soil moisture, a majority of the individual drought events producing this decline are predictable.

### Projections of warming air temperature impacts on southeastern Ethiopia

We conclude our analysis by taking a longer (1950–2014) look at southeastern Ethiopia climate conditions, combining gridded precipitation estimates and air temperatures values to approximate changes in soil moisture over longer periods than those covered by the CHIRPS. The basis of this approximation was the regression between annual soil moisture, rainfall, and air temperatures, which indicated strong relationships (CV R^2^ 0.88). We re-iterate that this topic will be revisited using multiple hydrologic models and a more detailed experimental design. Our point here, however, is to suggest i) that the recent hydrologic changes shown in Fig. 6 appear to be quite severe in the context of the observed changes in Ethiopia rainfall and air temperatures and ii) that warming air temperatures may already be contributing to substantial soil moisture reductions.

To represent rainfall on longer time scales we make use of a new FEWS NET resource, the Centennial Trends (CenTrends) dataset^31^; CenTrends covers Eastern Africa and benefits from 217 rainfall stations provided by the Ethiopian national meteorological agency. Standard error estimates from the CenTrends estimation process (kriging) indicates reasonable accuracies back to the 1950s.

The same station data have been used in the 1981–2014 CHIRPS version 2 and CenTrends version 1.0 datasets; the correlation between the October-September southeastern Ethiopia rainfall time series is 0.95. Fig. 8b shows 15-yr running averages of 1950–2014 standardized October-September CenTrends rainfall, standardized March-June CenTrends rainfall, and unstandardized GISS air temperatures. Both October-September and March-June rainfall decline substantially between the 1970s and 2000s. Air temperature increases have been slowly accelerating since the 1960s, in line with a 73 member/39 model ensemble of climate change simulations obtained from the Royal Netherlands Meteorological Institute (http://climexp.knmi.nl/). These air temperature anomalies (Fig. 8b) were adjusted (multiplied by 1.5), based on a regression between the observed time series and the mean of the climate change simulations.

The 1950–2014 correlation between the 15-yr observed and modeled air temperatures (red and dark red lines, Fig. 8b) is 0.96. The climate change simulations provided projections based on observed 1950–2005 greenhouse gases, aerosols and greenhouse gasses (the historic experiment^71^) (Taylor *et al.* ^71^), and 2006–2038 simulations from the 8.5 Wm^−2^ Representative Concentration Pathway experiment. This rapid warming scenario tracks closely with recent global emissions^72^. In both the model simulations and observations, the amount of warming since 1960 is quite large compared to the interannual standard deviation (~1 °C or 1.95 standardized deviations). By 2030, mean air temperatures are likely to rise another 0.4 °C (+~0.8 standardized deviation).

We can use our regression to estimate what this potential warming might entail (Fig. 8c). Between ~1970 and the 1990s, the influences of warming air temperatures may have been offset by increases in rainfall, resulting in little change in soil moisture. As rainfall decreased abruptly in the late 1990s and 2000s, soil moisture decreased as well, abetted by rising air temperatures. Overall, between the mid-1980s and late 2000s, 15-yr soil moisture anomalies decreased by ~1 standardized deviation—a substantial increase in aridity. About half of this decrease may be attributable to precipitation declines, and the VIC simulations suggest a similar magnitude of influence from rising air temperatures. If warming continues at the observed rate, as predicted by the climate change ensemble examined here, the mid-1980s to 2030 decrease in typical soil moisture conditions due to temperature influences alone might be ~−0.7 standardized deviation. A −0.7 sigma soil moisture anomaly is characteristic of the recent southeastern Ethiopia drought years (Fig. 6f).

### Summary and discussion

The CHIRPS dataset presented here has been designed for drought monitoring in places like Ethiopia—regions with complex topography, changing observation networks and deep convective precipitation systems that correspond reasonably well with CCD estimates. While broadly equivalent at seasonal time scales to gridded precipitation products like the GPCC (Figs 3 and 4, Table 2), the CHIRPS is updated frequently, and provides data at higher spatial and temporal resolutions. The CHIRPS incorporates satellite information in three ways: by using satellite means to produce high resolution precipitation climatologies, by using CCD fields to estimate monthly and pentadal precipitation anomalies, and by using satellite precipitation fields to estimate local distance decay functions (Fig. 2), guiding the interpolation process. Validation studies suggest that satellite-enhanced CHPclim compares favorably (Table 1) to the CRU and WordClim climatologies^19^. Constraining the CHIRP by the CHPclim reduces systematic estimation errors (Fig. 3), producing low MAE and bias statistics (Table 2). This fidelity, in terms of low frequency performance, may come at the cost of under-representing extremes, as suggested by recent CHIRPS evaluations in Mozambique^73^ and South America^74^. Future versions of the CHIRPS will explore less prescriptive estimation procedures.

Another issue identified in this study is the tendency for the CHIRPS to underestimate the variance in some places, like southwest North America (Fig. 4e), where the satellite-only estimates perform well, in terms of correlation (R=0.70), but heavily underestimate the variance. This may relate to specifics of the frontal precipitation systems characteristic of this region, as well as the fact that the TMPA 3B42 training data used in the CHIRP development process also performs relatively poorly over this region (Fig. 4f). On the other hand, the consistency of the CHIRP inputs and the anomaly-based interpolation process used to incorporate stations helps limit spurious excursions caused by changes in the satellite observation network or precipitation gauge networks.

While improvements to the CHIRPS process are already being developed, the current version 2.0 seems well suited to monitoring droughts in regions where CCD estimates relate reasonably well to observed precipitation systems. Our Ethiopia study provides a good example of CHIRPS utility. Because CHIRPS is produced in near real-time, and October-March southeastern Ethiopia soil moisture exhibits a strong lagged correlation with April-September conditions, October-March CHIRPS data can be used in conjunction with hydrologic models like the VIC to monitor conditions and make successful forecasts (Fig. 8a) that capture most of the recent extreme drought events.

These forecasts combine skill from the inherent persistence of soil moisture states with skill accruing from persistent La Niña-like climate disruptions (Fig. 7). These patterns associate warming in the western Pacific and cooling in the eastern Pacific with increased drought over eastern East Africa, and the exceptional strength of this SST gradient is thought to contribute to the declining boreal spring rains in that region^32,48^. The SST warming patterns associated with southeastern Ethiopia drying (Fig. 7b) highlight the same warming regions identified by ENSO-residual trend analyses^75,76^ or ENSO-residual empirical orthogonal function analysis^77^. The regions associated with dry southeastern Ethiopia weather have warmed rapidly, in line with climate change projections^77^, especially when paired with La Niña-like cool central Pacific SST, can produce southeastern Ethiopia drying, and may continue to do so, unless eastern Pacific SST begins warming in accordance with most climate change simulations, which predict a wetter East Africa^78^. Climate change projections of precipitation for Ethiopia are quite diverse, but on average predict little change at the national level^65^. New paleo-climate evidence, however, based on sediment records from the Gulf of Aden, indicate long-term links between global warming and hotter, drier conditions in East Africa^79^, in line with observations of boreal spring rainfall^31^, the west Pacific SST gradient^32,48^, and the West Pacific Warming Mode^56^.

While temperature data are sparse, substantial changes in temperature, on the other hand, have been observed in Ethiopia, with estimates of warming rates around +0.3 °C per decade^66,80,81^. The VIC modeling results presented suggest that at annual time scales over the large southeastern Ethiopia domain, the *aggregate* effect of this warming may be affecting water availability, substantially reducing runoff (Fig. 6f) and soil moisture (Figs 6g and 8c) in a chronically food-insecure region (Fig. 5a) likely to have 32 million people by 2020 and 62 million people by 2050 (ref. 47). Building the close correspondence between annual simulated southeastern Ethiopia VIC soil moisture values and regression estimates based on annual air temperatures and rainfall, we suggest that a ~+2 °C warming of air temperatures may soon (~2030) reduce average southeastern Ethiopia soil moisture conditions by more than −0.7 of a standardized deviation. These results need to be corroborated with more hydrologic models.

In line with recent global assessments^82^, we have potentially identified substantial temperature contributions that have enhanced the effects of drought in southeastern Ethiopia, contributing perhaps 50 and 30% of the 1999–2014 reductions in runoff and soil moisture. As shown in Trenberth *et al.* ^82^, precipitation must be taken into account when evaluating the drought impacts of temperature, and use of datasets like the CHIRPS should aid in this task. ‘Routine’ monitoring, like driving the VIC with real-time precipitation, can produce soil moisture estimates indicative of future outcomes (Fig. 8a)^40,43^. These types of powerful yet relatively simple forecasts may help us better manage 21st century climate variability, thereby better adapting to our changing climate.

While more work needs to be done to verify the simulation results presented here, and the CHIRPS and CHPclim algorithms will continue to be improved, this paper has described the CHIRPS algorithm, presented some promising validation results, and provided a motivating example application. One substantial weakness of the current CHIRPS algorithm is the lack of uncertainty information provided by the inverse distance weighting algorithm used to blend the CHIRP data and station data. We are currently exploring more rigorous geostatistical models (related to kriging^83,84^) and plan to use such frameworks to provide standard error fields in future CHIRPS releases. Ongoing research is also exploring mean bias errors with independent validation datasets. This work should lead to improved CHPclim fields, which would improve skill by better capturing the geographic variability of rainfall.

The U.S. Geological Survey (USGS)/Climate Hazards Group science team is also developing tools that can work with the CHIRPS, or other gridded precipitation datasets, to calculate the crop Water Requirement Satisfaction Index^85,86^, blend in situ observations with the satellite precipitation fields, and analyze climate trends and anomalies. These tools (GeoWRSI and GeoCLIM) and the CHIRPS dataset are available at http://chg.ucsb.edu/tools. The CHIRPS can also be viewed dynamically using the suite of visualizations available at earlywarning.usgs.gov. Climate variability can have a profound impact on the economies of countries like South Africa^87^ or Ethiopia^65^, and developing national capacities to deal with climate variability will be a critical component of climate adaptation^88^. National meteorological agencies can begin with precipitation estimates like CHIRPS, TARCAT, RFE2, or ARC gridded precipitation products, add their station data using tools like GeoCLIM^89^ or the International Research Institute’s Enhancing National Climate Time Series (ENACTS) software^90^, and develop information products that may improve disaster mitigation and climate adaption. By taking advantage of the processing involved in producing the CHIRPS, and adding value to the dataset by incorporating more in situ observations and local knowledge regarding risks and exposure, we hope that more scientists will be able to provide valuable climate services and environmental data, leading to improved adaptation and disaster mitigation.

## Additional Information

**How to cite this article:** Funk, C. *et al.* The climate hazards infrared precipitation with stations—a new environmental record for monitoring extremes. *Sci. Data*. 2:150066 doi: 10.1038/sdata.2015.66 (2015).

## Supplementary Material



## Figures and Tables

**Table 1:** CHPclim, CRU, and Worldclim validation results.

	**Colombia**	**Afghanistan**	**Ethiopia**	**Sahel**	**Mexico**
Stations	194	22	76	28	1,814
CHPclim-MBE	5%	3%	4%	0%	−2%
CRU-MBE	−6%	−28%	−4%	16%	2%
Worldclim-MBE	−6%	−17%	0%	−16%	−2%
CHPclim-MAE	18%	25%	10%	10%	30%
CRU-MAE	28%	57%	24%	21%	31%
Worldclim-MAE	19%	52%	21%	22%	23%
CHPclim-R^2^	84%	53%	91%	93%	65%
CRU-R^2^	58%	18%	68%	87%	60%
Worldclim-R^2^	82%	18%	72%	86%	78%

**Table 2:** 2000–2010 GPCC wet season total comparison statistics

**source**	**semi-Global Correlation**	**Africa Correlation**	**USA Correlation**
CFS	0.52	0.41	0.73
CHIRP	0.44	0.38	0.55
CHIRPS	0.67	0.56	0.89
CPCU	0.69	0.6	0.85
ECMWF	0.49	0.4	0.69
TMPA 3B42 RT7	0.39	0.3	0.55
MAE values represent mm over the 3 month wet season.			
The bias values were calculated as abs(1-mean(source)/mean(GPCC)).			

**source**	**semi-Global mean MAE**	**Africa mean MAE**	**USA mean MAE**
CFS	153	162	72
CHIRP	99	90	61
CHIRPS	82	79	37
CPCU	100	114	38
ECMWF	131	122	68
TMPA 3B42 RT7	157	139	137
MAE values represent mm over the 3 month wet season.			
The bias values were calculated as abs(1-mean(source)/mean(GPCC)).			

**source**	**semi-Global mean Bias**	**Africa mean Bias**	**USA mean Bias**
CFS	0.41	0.43	0.28
CHIRP	0.26	0.25	0.15
CHIRPS	0.21	0.22	0.11
CPCU	0.22	0.3	0.12
ECMWF	0.34	0.34	0.22
TMPA 3B42 RT7	0.44	0.39	0.54
MAE values represent mm over the 3 month wet season.			
The bias values were calculated as abs(1-mean(source)/mean(GPCC)).			
